# Creating qualitative datasets in geoarchaeology: An easy-applicable description template for archaeological thin section analysis using Stoops 2003

**DOI:** 10.1016/j.mex.2022.101663

**Published:** 2022-03-08

**Authors:** Diana Marcazzan, Sarah Ann Meinekat

**Affiliations:** Institute for Archaeological Sciences, University of Tübingen, Germany

**Keywords:** Archaeology, Geoarchaeology, Micromorphology, Description template, Qualitative data

## Abstract

Micromorphological thin section analysis is a powerful method in geoarchaeology to study deposits at archaeological sites. The approach is largely borrowed from soil science and relies on standard descriptive terminology established by [9] and others. Within archaeological micromorphology, we encounter two main issues with thin section description: (1) Anthropogenic deposits are highly variable when compared to purely natural deposits and soils and (2) many practitioners of archaeological micromorphology come from a range of backgrounds and experiences. Therefore, despite the use of standardized terminology, the qualitative nature of thin section description results in a high degree of variation amongst practitioners in the description and documentation of thin sections, particularly amongst beginners in the field.

Here, we propose:

• A template that can help to understand and remember the terminology, so that it can be an easy-to-use tool for beginners.
• A template with better inclusion of anthropogenic material and features to better fit the needs of archaeological sites.
• A way of documenting data from a thin section analysis, that results in understandable, reproducible, and shareable datasets that can be easily integrated with databases.

## Graphical abstract

**Figure:**
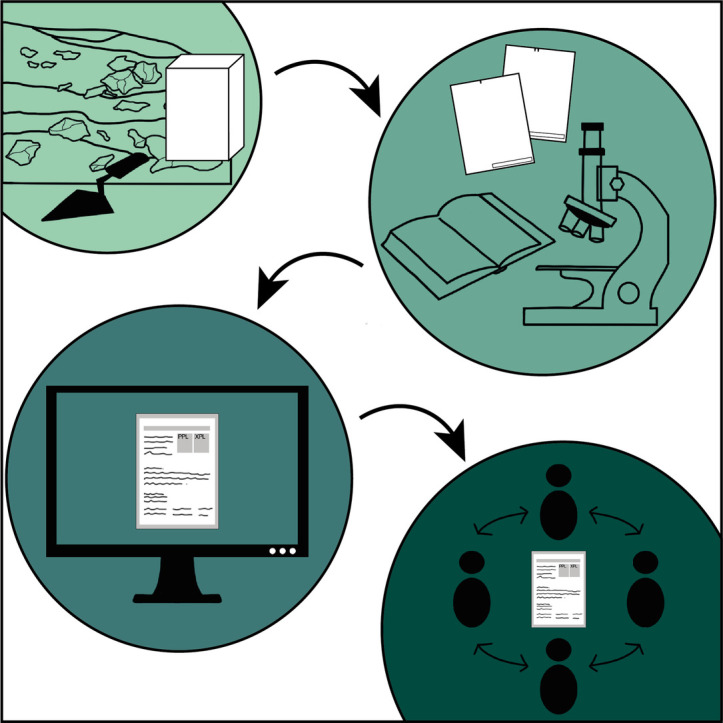

Specifications Table

**Table:** 

Subject Area:	*Earth and Planetary Sciences*
More specific subject area:	*Geoarchaeology*
Method name:	*Archaeological Thin Section Analysis*
Name and reference of original method:	*Guidelines for Analysis and Description of Thin Sections* *Stoops, G. (2003, 2021). Guidelines for Analysis and Description of Soil and Regolith Thin Sections. John Wiley & Sons.*
Resource availability:	*Microsoft Office (file in Supplementary Material)*

## Introduction and background

Soil and sediment micromorphology is a fast-advancing technique in geoarchaeology that has become established through the past decades [1], [2], [3], [4], [5], [6], [7], [8], [9]. It derives from Soil Sciences and is based on the micro-analysis of undisturbed and orientated sediment/soil samples.

Issues arise within the application of micromorphology in (geo-)archaeology. Firstly, archaeologists have different backgrounds, and often not in the geosciences. Thus, the plethora of specialized terms used in manuals is difficult to understand and remember for beginners in the field. The variety of experiences and backgrounds can lead to variable results and identifications [9]. Secondly, soil and sediment micromorphology is mostly a qualitative method, making it difficult to ensure that results are comparable, reproducible, and published using high-quality documentation. For this reason, one of the main aims of researchers has always been to promote the use of standard terminology and comprehensive publishing practices [1], [2], [4], [6], [8], [9].

To address these issues, there have been repeated approaches to create templates and data forms for recording thin section data (for example the back cover of Bullock et al. 1985) In recent years, geoarchaeology working groups at different universities have been especially active in this matter, understanding the need to integrate soil science and archaeological components for thin section analysis within geoarchaeology (see below). Unfortunately, such lab protocols are not commonly published and therefore not easily accessible or citable to peers.

While manuals like [1], [9] provide the geoscientific background and common terminology, the descriptive method for data recording in soil and sediment micromorphology is often not consistently used and can be insufficient for archaeological contexts. A strong focus has been on how we present and publish the micromorphological data and interpretations in papers, to peers, as well as to a broader audience [8], [9], [10]. In contrast, less has been published on how to collect and create datasets with qualitative data as the first step in thin section analysis.

Our contribution focuses on how to create micromorphology datasets systematically, utilizing some of the standard background literature and terminology, to make the data comprehensible, reproducible, comparable, and shareable. The template offers a first step in the analysis of archaeological thin sections and creates a base upon which subsequent interpretational steps can be built.

## Method details

### Classic thin section analytical steps

**Fig. 1:**
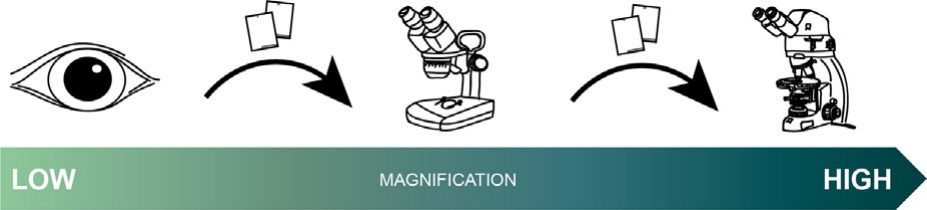
Thin section analysis steps. Naked-eye, stereomicroscope, and petrographic microscope observation.

For thin section analysis, it is a common procedure to move from low to high magnification (Fig. 1) [6]. The first observations are made with the naked eye, oftentimes supported with the use of a light table. For documentation and first observations, we also scan the thin sections in low and high resolution, using a flatbed, and a film scanner [11]**.** The thin section can then be analysed with a stereomicroscope. After a first observation with a stereomicroscope, the analysis under high magnification is performed with a petrographic microscope for a further component and mineral identification. Analyses are done under plane-polarized (PPL) and crossed-polarized light (XPL). Additional insights can be gained from the use of other types of light, such as oblique incident light (OIL) and blue light fluorescence.

The analysis follows geological criteria described in [9] Chapters 4–8, ranging from Fabric; Voids, Aggregates, Microstructure; Mineral and Organic Constituents; Groundmass; to Pedofeatures. In chapter 9, first tips on how to collect thin section data are given. Here, our description template expands the descriptive details and adapts the description to better suit the needs of archaeological settings.

### Description template - steps

One of the main goals with the creation of the template (Supplementary information SM2) was to ease the use of the specialized terminology. The template contains the criteria listed by [9] with the terms in drop-down menus. These menus remind the analyst of what to look for, thereby guiding them through the analysis and aiding in the creation of a thin section description dataset.

The template also has space for notes and remarks at all observation steps to allow for the addition of more detailed comments, ideas, and clarification. Archaeology-specific criteria were added to complement the description.

**Fig. 2:**
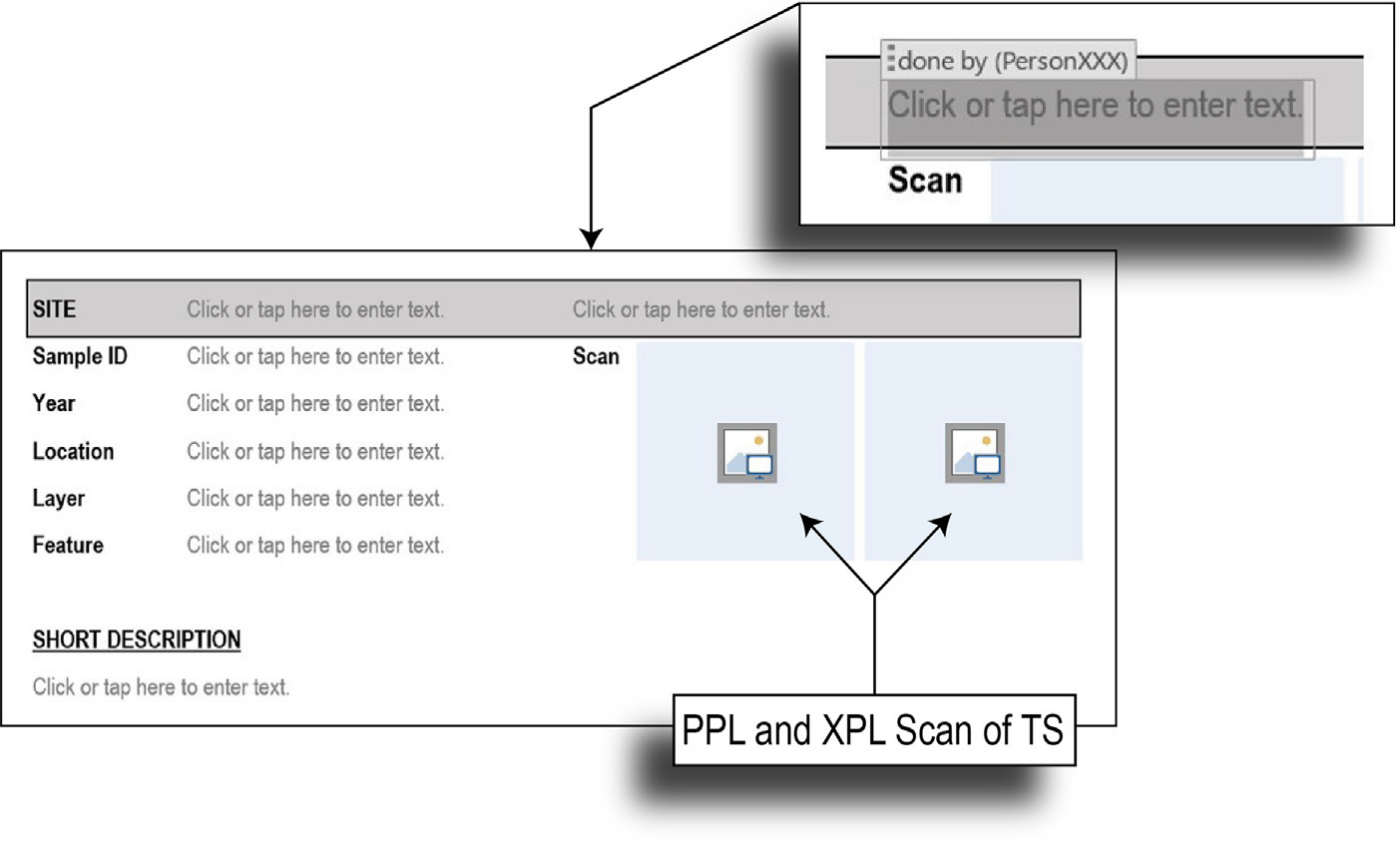
Step 1 - General sample information.
*Step 2* - After this, the microscopic analysis details follow (Fig. 3). At archaeological sites, the sampling strategy is often based on sampling different layers with one sample, covering a specific transition or interface. This strategy means that one archaeological thin section often includes several microstratigraphic units (micro-units). To address this issue, we added a section to specify the micro-unit (Fig. 3). The following details should be added for all micro-units. The micro-units may also be indicated in the scans. If the microfacies concept [12,13] is applied, the microfacies type can be indicated next. Please note that often the microfacies classification is done after initial description of the thin sections and might therefore be added later.

*Step 1* - First, basic information, such as site name, sample ID, year, analyst, together with archaeology specific information, such as the excavation unit, layer, and/or feature are noted (Fig. 2). Scans in PPL and XPL can be added. A short description of naked-eye observations, or field particularities also has space here (Fig. 2).

**Fig. 3:**
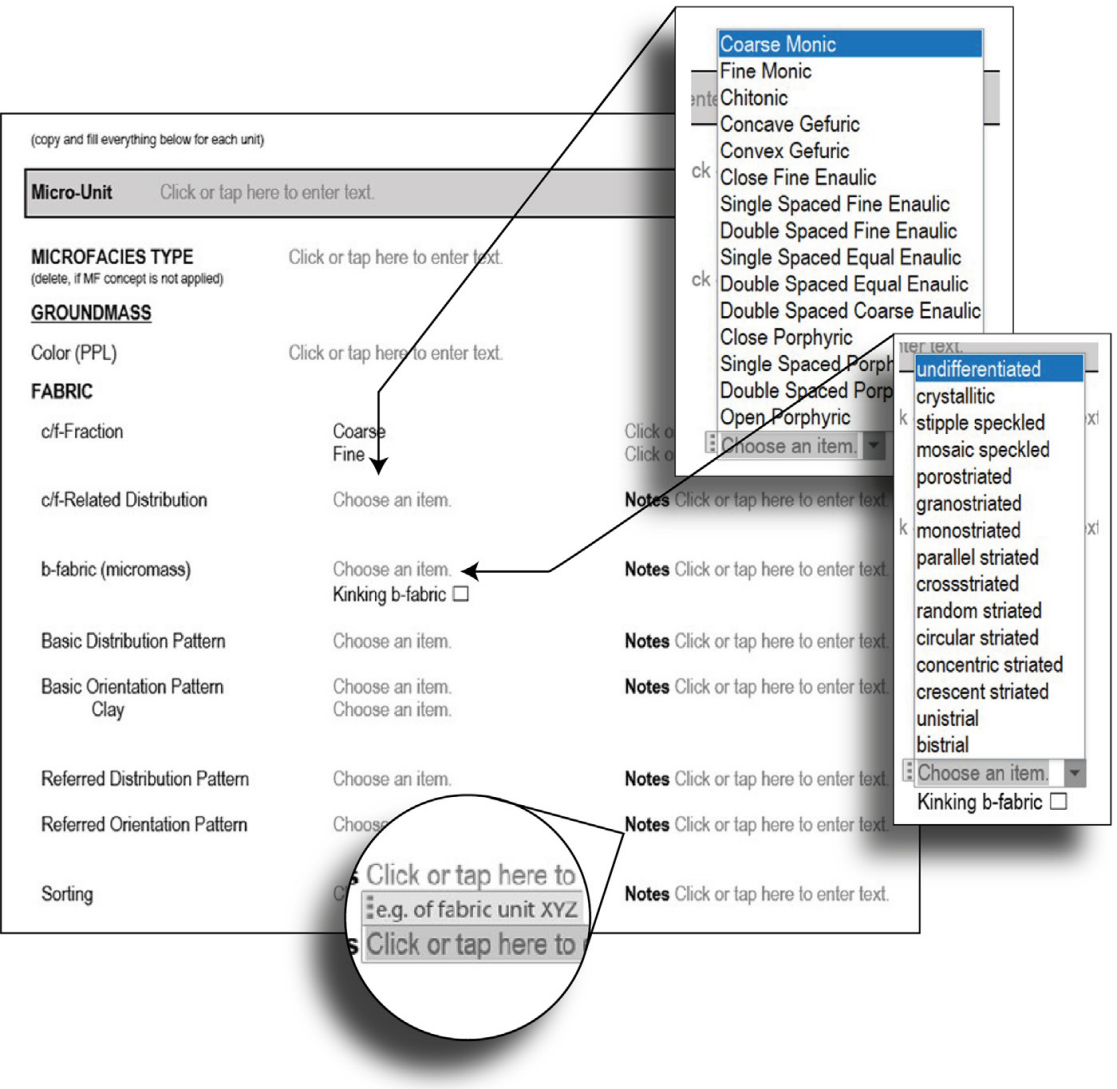
Step 2 - Micro-Unit analysis, groundmass details.

For each micro-unit, the following parts are described: Groundmass, main components, pedofeatures, and interface to upper unit. The description starts with analysis of the groundmass and details on the fabric (Fig. 3).

Here, the first special feature of the template appears: the specialized terms from Stoops (2003), [9] are visible and selectable in a drop-down menu (Fig. 3). Notes can be added on the right-hand side. The notes field also offers suggestions of what kind of information might be helpful to add.

**Fig. 4:**
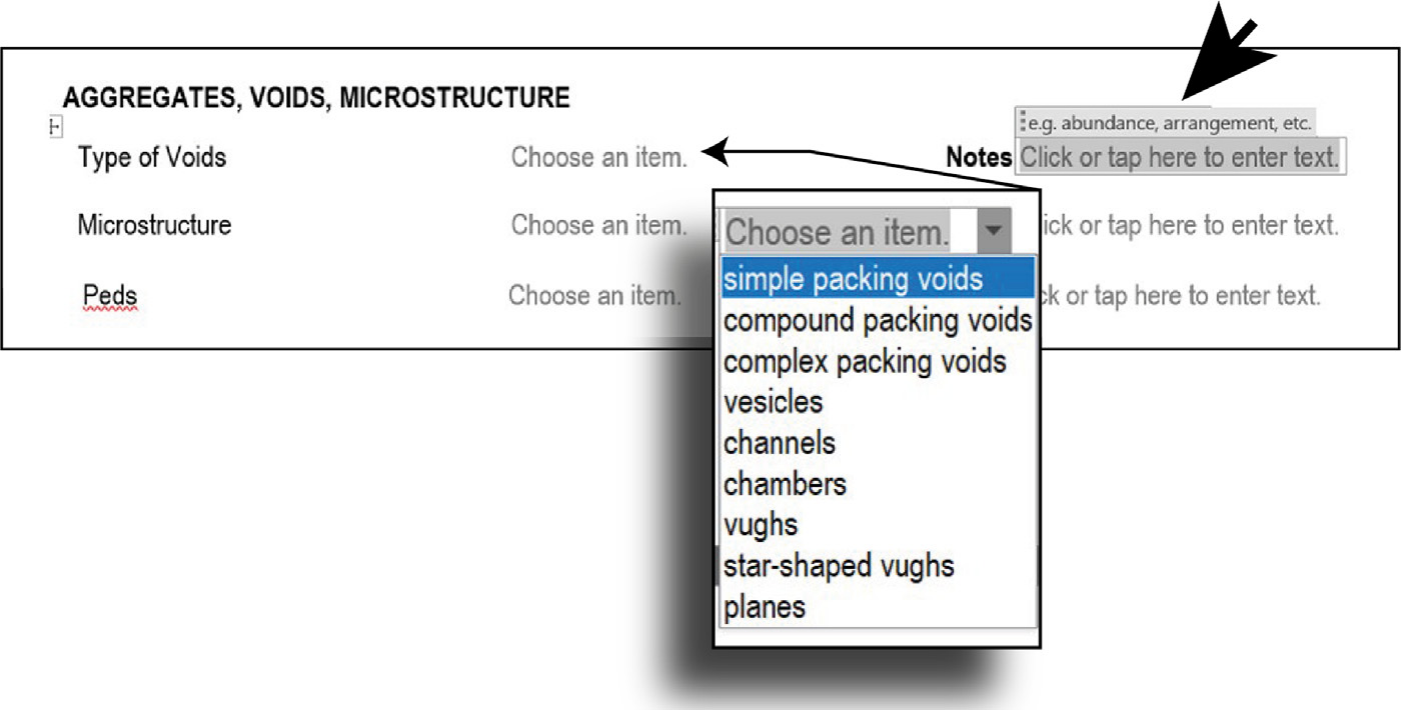
Step 2 - Micro-Unit analysis, aggregates, voids, and microstructure details.

In the next part (Fig. 4), aggregates, voids, and microstructure are described, including similar features in the template as before, such as drop-down menus, and note suggestions.

**Fig. 5:**
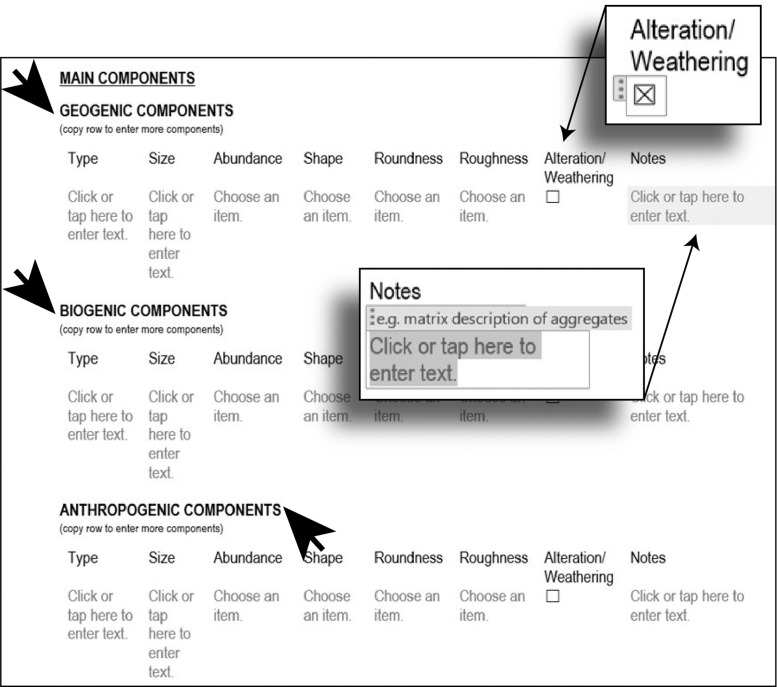
Step 2 - Micro-Unit analysis, component details.

Then, the main components are identified and analysed (Fig. 5). Three main categories, divided by the depositional agents, can be described (geogenic, biogenic, and anthropogenic). Each component has a row in which details are noted, such as the presence of weathering that can be indicated by simple box-checking. If further clarification is necessary about a specific component, additional information can be added in the notes section.

**Fig. 6:**
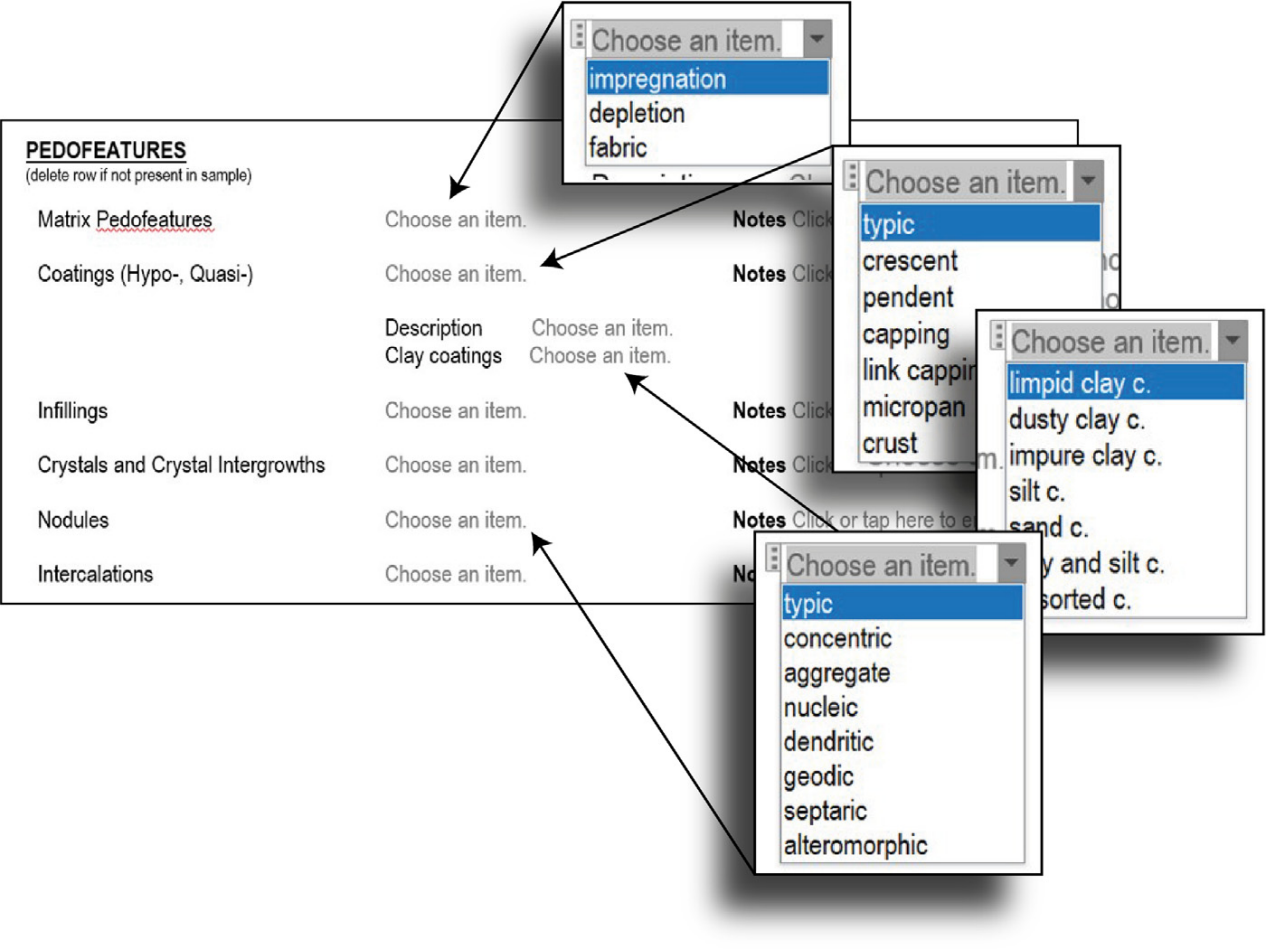
Step 2 - Micro-Unit analysis, pedofeatures details.

Pedofeatures are described in the next part (Fig. 6). Here, the number of highly specific terms is highlighted again. The drop-down menus guide the analyst through the plethora of terms.

**Fig. 7:**
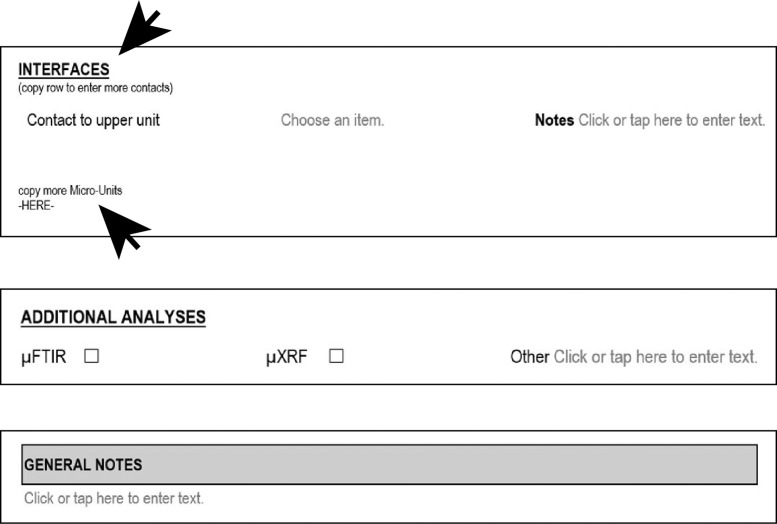
Step 2 - Micro-Unit analysis, interfaces; Step 3 - Additional micro-units; Step 4 - Additional analyses and notes.

The template also contains a section for describing interfaces (Fig. 7). This is especially important for archaeological thin sections, and those that contain several micro-units.

*Step 3* - After the section on interfaces, further micro-unit descriptions can be added (Fig. 7). If a thin section does not contain a certain characteristic, the respective row can be deleted. Whenever more space or rows are needed at a certain part, those can be copied and pasted as indicated.
*Step 4* - At the end of the template, it can be noted if and what kind of additional analyses have been or will be performed on the thin section (Fig. 7). µFTIR and µXRF, which are the most common additional analyses performed for our laboratory, were added with checkboxes. If something else, e. g. SEM was performed, it can be noted in the “Others” section.

At the bottom, we again added a box for notes (Fig. 7). Space in the small note boxes is limited, and this field allows for longer comments and ideas to be recorded. It is also a place where previous published works on the thin section or the site can be added for reference and links.

## Value

With this template, we create a dataset that accompanies each thin section. It helps to keep the descriptive analysis separate from subsequent interpretative steps. Currently, many of us instead have a mix of various notes, tables, and micrographs from all stages of the process. While this mixture is a common and valid way of collecting thin section data it is complicated to integrate and use such data with:

(1) Digital databases and modern data archiving.
(2) Sharing data with peers and third parties.
(3) Student training.
(4) Current open access requirements by journals.

(1) Data archiving and digitization have become increasingly important in many fields and ours is no different. It is imperative to acknowledge that we as scientists are responsible for our data, their archiving, and their accessibility. Thus, integrating thin section data with digital databases is one of our main focuses. Datasets created using this template can be more easily integrated with databases and accompany each thin section.

(2) In (geo-)archaeology we often work with state offices and other third parties. These groups sometimes require interim results or proof of work before publishing results and interpretations. The dataset provides us with a standardized form we can quickly share with involved parties.

(3) Micromorphology is a method that is experience-based. Learning the method can be overwhelming. The drop-down menus within the template help to overcome this initial issue and is a useful training tool. Beginners in the field or those returning to it after some time will be reminded of what to look for, thereby guiding them through the basic analysis. Analysts working through the steps are less likely to only look for what they are interested in, disregarding other, perhaps important aspects. The template helps to avoid a one-sided, predisposed approach to the analysis. Furthermore, it also helps to familiarize beginners with the standard terminology. The template also helps to differentiate between description (i.e., information going into the dataset) and interpretation (i.e., inferences based on the information in the dataset).

(4) Data sharing and data accessibility in the times of open access publishing is changing the way we maintain our data. Many journals ask for the base data of a paper to be accessible. The datasets should not ``be'' the paper, but rather the ``raw data'' that the paper -and interpretations- are based on. The actual raw data will of course always be the thin section but sharing the slides themselves is obviously not feasible. High-resolution film scans, that come closest to the slide itself [11], will overwhelm any online system due to their sheer data size. The dataset created by completing this template with the core data, presents an alternative way of sharing our “raw data” that easily fits in any sharing system.

## Conclusion

The template provides a useful guiding framework during the analytical process but it is not possible to cover every possible situation that may be present within a thin section. Thus, this framework can and should be adapted to each site and project through additions or deletions of components, the addition of different sections, and the continued use of the notes boxes. Additionally, the dataset produced through completion of the template must be accompanied by visual documentation of the thin section in the form of scans and photomicrographs.

Even though, or rather because our data are mostly qualitative, we want to promote a way of collecting data that is a combination of high-quality documentation, comparable and systematic results, and shareable and reproducible datasets. (Semi-)quantitative data, as well as data from other techniques and methods [10], can easily be integrated into the template. The template gives us a datasheet that we can share and a way to be responsible for our data, also in the long term [14]. The framework that the template provides leads to a more streamlined analysis process.

The dataset contains but the base data of the thin sections. Interpretation is still required and relies on skilled and well-trained micromorphology experts.

Lastly, it is our hope that by publishing our working group's template, we encourage other groups to share their protocols and templates.

## Declaration of Competing Interest

The authors declare that they have no known competing financial interests or personal relationships that could have appeared to influence the work reported in this paper.

## Appendix Supplementary materials

Image, application 1
