# Supplementary material for: Creating qualitative datasets in geoarchaeology: An easy-applicable description template for archaeological thin section analysis using Stoops 2003
Source: MethodsX. 2022 Mar 8;9:101663. doi: 10.1016/j.mex.2022.101663 (PMC8943325; doi:10.1016/j.mex.2022.101663)
Supplement: Supplementary file 1 [file mmc1.docx]

SM1

Example of the template used for analysis of archaeological thin sections, example of Fumane Cave (Italy) and Quebrada Jaguay-280 (Peru).


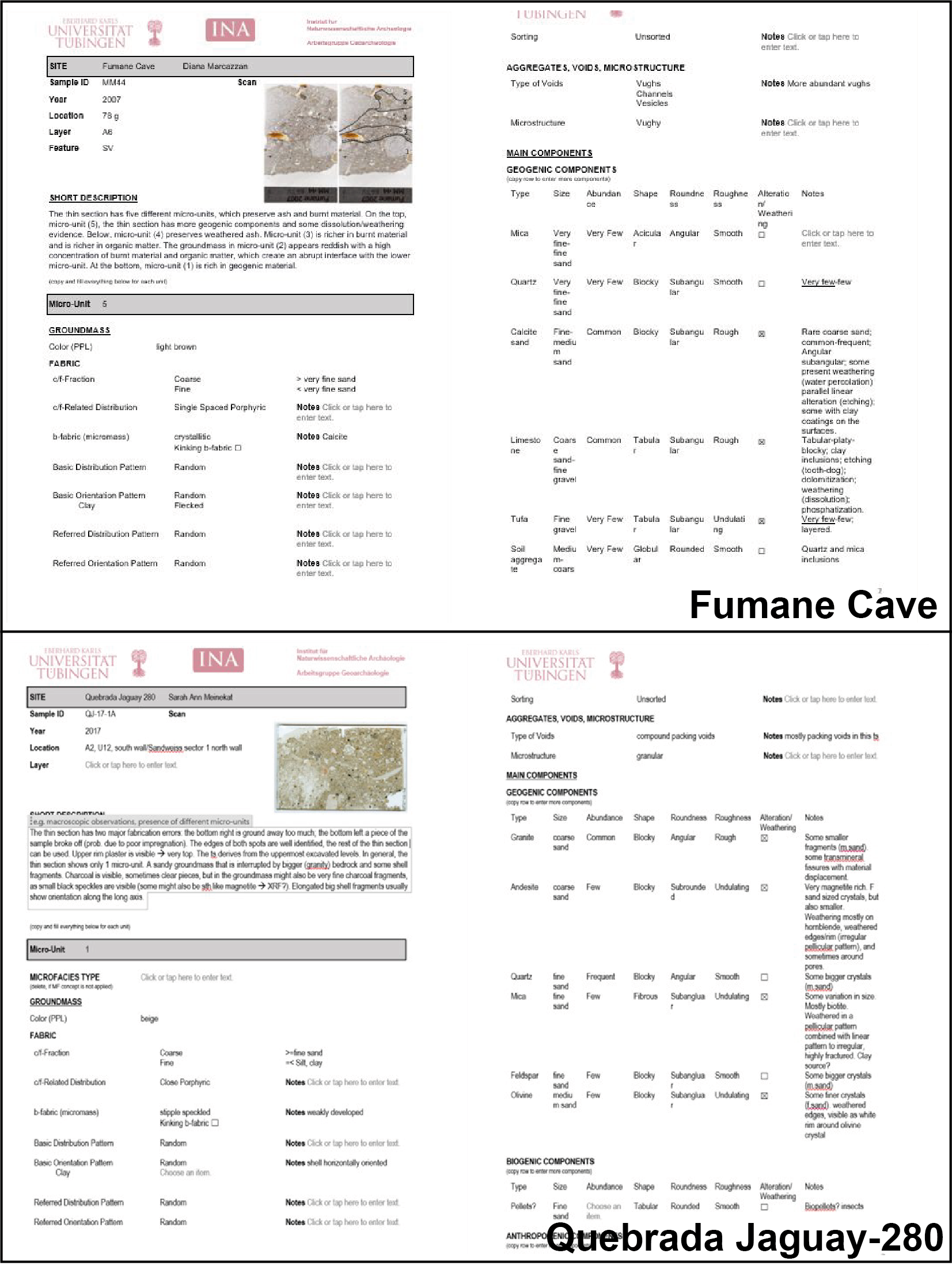


SM2

Word document template file

**SITE** Click or tap here to enter text. Click or tap here to enter text.

**Sample** **ID** Click or tap here to enter text. **Scan
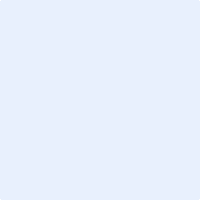

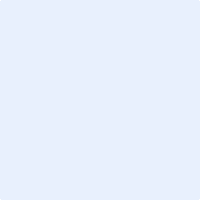
**

**Year** Click or tap here to enter text.

**Location** Click or tap here to enter text.

**Layer** Click or tap here to enter text.

**Feature** Click or tap here to enter text.

**SHORT DESCRIPTION**

Click or tap here to enter text.

(copy and fill everything below for each unit)

**Micro-Unit** Click or tap here to enter text.

**MICROFACIES TYPE** Click or tap here to enter text.
(delete, if MF concept is not applied)

**GROUNDMASS**

Color (PPL) Click or tap here to enter text.

**FABRIC**

| c/f-Fraction | Coarse  Fine | Click or tap here to enter text.  Click or tap here to enter text. |
| --- | --- | --- |
| c/f-Related Distribution | Choose an item. | **Notes** Click or tap here to enter text. |
| b-fabric (micromass) | Choose an item.  Kinking b-fabric | **Notes** Click or tap here to enter text. |
| Basic Distribution Pattern | Choose an item. | **Notes** Click or tap here to enter text. |
| Basic Orientation Pattern  Clay | Choose an item.  Choose an item. | **Notes** Click or tap here to enter text. |
| Referred Distribution Pattern | Choose an item. | **Notes** Click or tap here to enter text. |
| Referred Orientation Pattern | Choose an item. | **Notes** Click or tap here to enter text. |
| Sorting | Choose an item. | **Notes** Click or tap here to enter text. |

**AGGREGATES, VOIDS, MICROSTRUCTURE**

| Type of Voids | Choose an item. | **Notes** Click or tap here to enter text. |
| --- | --- | --- |
| Microstructure | Choose an item. | **Notes** Click or tap here to enter text. |
| Peds | Choose an item. | **Notes** Click or tap here to enter text. |

**MAIN COMPONENTS**

**GEOGENIC COMPONENTS**
(copy row to enter more components)

| Type | Size | Abundance | Shape | Roundness | Roughness | Alteration/ Weathering | Notes |
| --- | --- | --- | --- | --- | --- | --- | --- |
| Click or tap here to enter text. | Click or tap here to enter text. | Choose an item. | Choose an item. | Choose an item. | Choose an item. |  | Click or tap here to enter text. |

**BIOGENIC COMPONENTS**
(copy row to enter more components)

| Type | Size | Abundance | Shape | Roundness | Roughness | Alteration/ Weathering | Notes |
| --- | --- | --- | --- | --- | --- | --- | --- |
| Click or tap here to enter text. | Click or tap here to enter text. | Choose an item. | Choose an item. | Choose an item. | Choose an item. |  | Click or tap here to enter text. |

**ANTHROPOGENIC COMPONENTS**
(copy row to enter more components)

| Type | Size | Abundance | Shape | Roundness | Roughness | Alteration/ Weathering | Notes |
| --- | --- | --- | --- | --- | --- | --- | --- |
| Click or tap here to enter text. | Click or tap here to enter text. | Choose an item. | Choose an item. | Choose an item. | Choose an item. |  | Click or tap here to enter text. |

**PEDOFEATURES**(delete row if not present in sample)

| Matrix Pedofeatures | Choose an item. | **Notes** Click or tap here to enter text. |
| --- | --- | --- |
| Coatings (Hypo-, Quasi-) | Choose an item.  Description Choose an item.  Clay coatings Choose an item. | **Notes** Click or tap here to enter text. |
| Infillings | Choose an item. | **Notes** Click or tap here to enter text. |
| Crystals and Crystal Intergrowths | Choose an item. | **Notes** Click or tap here to enter text. |
| Nodules | Choose an item. | **Notes** Click or tap here to enter text. |
| Intercalations | Choose an item. | **Notes** Click or tap here to enter text. |

**INTERFACES**(copy row to enter more contacts)

| Contact to upper unit | Choose an item. | **Notes** Click or tap here to enter text. |
| --- | --- | --- |

copy more Micro-Units
-HERE-

**ADDITIONAL ANALYSES**

µFTIR  µXRF  Other Click or tap here to enter text.

**GENERAL NOTES**

Click or tap here to enter text.
